# Supplementary figures and images for: Orai3 and Orai1 mediate CRAC channel function and metabolic reprogramming in B cells
Source: eLife. 2023 Feb 21;12:e84708. doi: 10.7554/eLife.84708 (PMC9998091; doi:10.7554/eLife.84708)

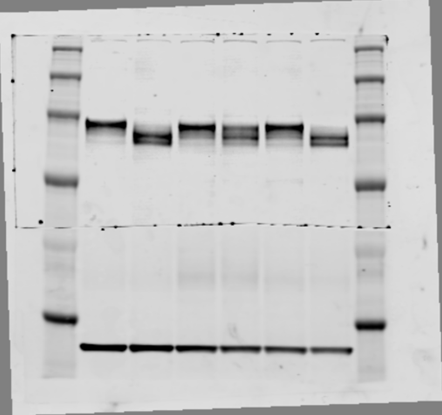

Supplement: Figure 6—source data 2. [file elife-84708-fig6-data2.zip › Figure 6D.tif]

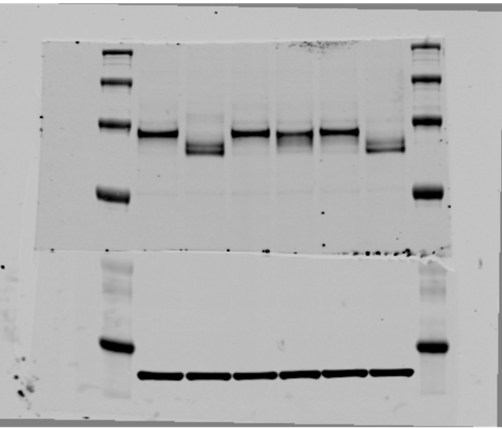

Supplement: Figure 6—source data 2. [file elife-84708-fig6-data2.zip › Figure 6F.tif]

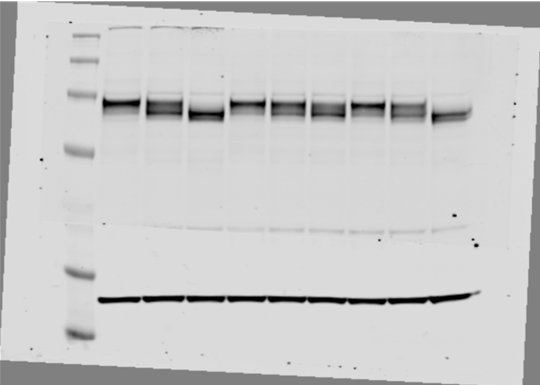

Supplement: Figure 6—figure supplement 1—source data 2. [file elife-84708-fig6-figsupp1-data2.zip › Figure 6-Figure Supplement 1A NFAT1.tif]

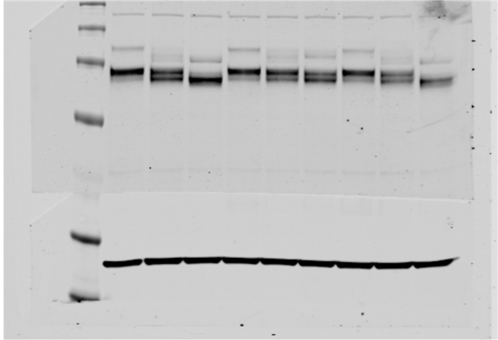

Supplement: Figure 6—figure supplement 1—source data 2. [file elife-84708-fig6-figsupp1-data2.zip › Figure 6-Figure Supplement 1A NFAT2.tif]

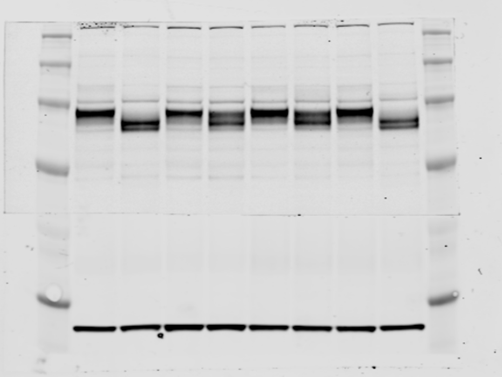

Supplement: Figure 6—figure supplement 1—source data 2. [file elife-84708-fig6-figsupp1-data2.zip › Figure 6-Figure Supplement 1B.tif]

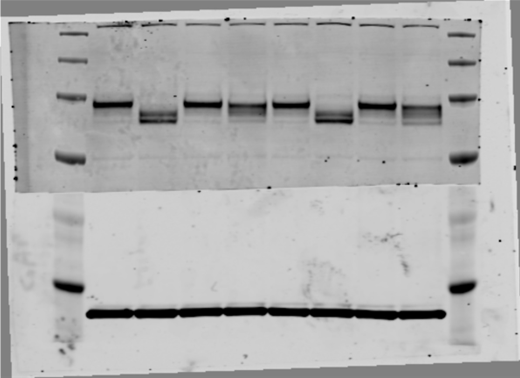

Supplement: Figure 6—figure supplement 1—source data 2. [file elife-84708-fig6-figsupp1-data2.zip › Figure 6-Figure Supplement 1C.tif]

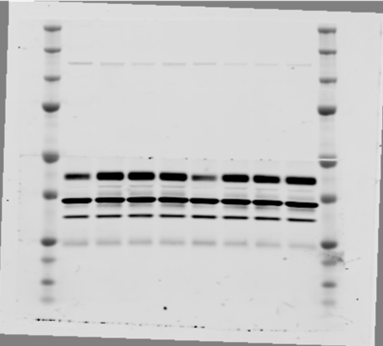

Supplement: Figure 8—figure supplement 1—source data 2. [file elife-84708-fig8-figsupp1-data2.zip › Figure 8-Figure Supplement 2A GAPDH MCU.tif]

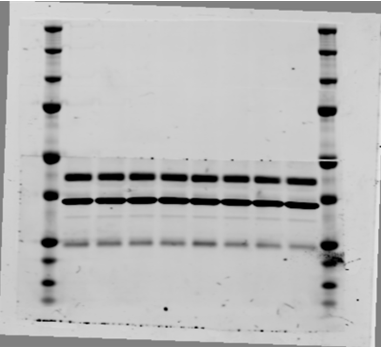

Supplement: Figure 8—figure supplement 1—source data 3. [file elife-84708-fig8-figsupp1-data3.zip › Figure 8-Figure Supplement 2A CREB.tif]

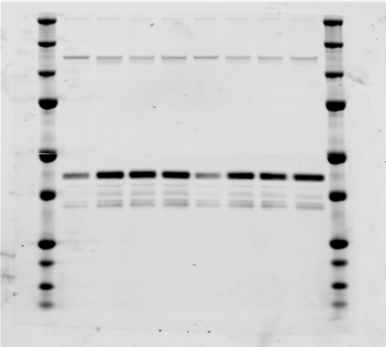

Supplement: Figure 8—figure supplement 1—source data 3. [file elife-84708-fig8-figsupp1-data3.zip › Figure 8-Figure Supplement 2A pCREB.tif]

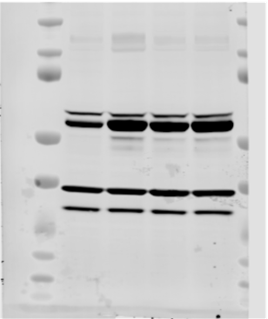

Supplement: Figure 8—figure supplement 1—source data 4. [file elife-84708-fig8-figsupp1-data4.zip › Figure 8-Figure Supplement 2B.tif]

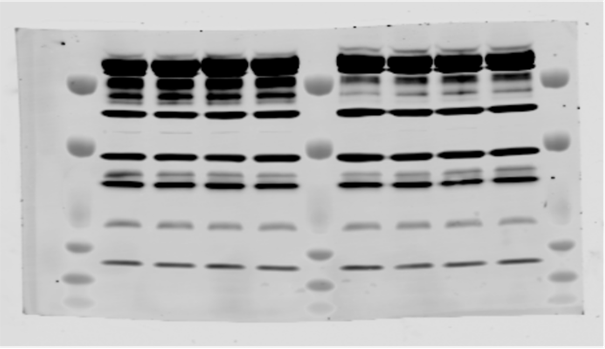

Supplement: Figure 8—figure supplement 1—source data 6. [file elife-84708-fig8-figsupp1-data6.zip › Figure 8-Figure Supplement 2C GAPDH.tif]

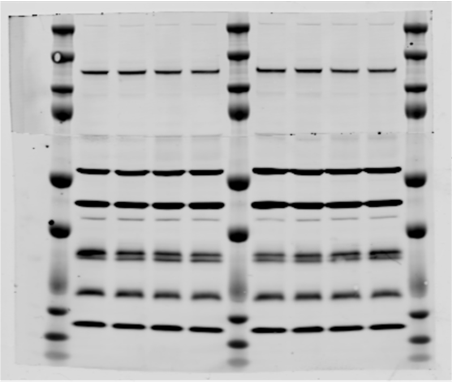

Supplement: Figure 8—figure supplement 1—source data 7. [file elife-84708-fig8-figsupp1-data7.zip › Figure 8-Figure Supplement 2D ETC.tif]

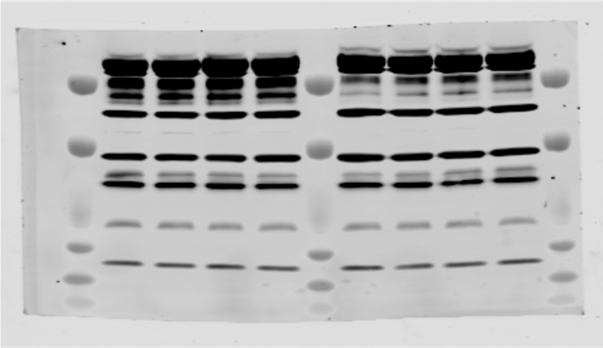

Supplement: Figure 8—figure supplement 1—source data 7. [file elife-84708-fig8-figsupp1-data7.zip › Figure 8-Figure Supplement 2D GAPDH.tif]
